# ING3 Is Essential for Asymmetric Cell Division during Mouse Oocyte Maturation

**DOI:** 10.1371/journal.pone.0074749

**Published:** 2013-09-16

**Authors:** Shinnosuke Suzuki, Yusuke Nozawa, Satoshi Tsukamoto, Takehito Kaneko, Hiroshi Imai, Naojiro Minami

**Affiliations:** 1 Laboratory of Reproductive Biology, Graduate School of Agriculture, Kyoto University, Kyoto, Japan; 2 Laboratory Animal and Genome Sciences Section, National Institute of Radiological Sciences, Chiba, Japan; 3 Institute of Laboratory Animals, Graduate School of Medicine, Kyoto University, Kyoto, Japan; Institute of Zoology, Chinese Academy of Sciences, China

## Abstract

ING3 (inhibitor of growth family, member 3) is a subunit of the nucleosome acetyltransferase of histone 4 (NuA4) complex, which activates gene expression. ING3, which contains a plant homeodomain (PHD) motif that can bind to trimethylated lysine 4 on histone H3 (H3K4me3), is ubiquitously expressed in mammalian tissues and governs transcriptional regulation, cell cycle control, and apoptosis via p53-mediated transcription or the Fas/caspase-8 pathway. Thus, ING3 plays a number of important roles in various somatic cells. However, the role(s) of ING3 in germ cells remains unknown. Here, we show that loss of ING3 function led to the failure of asymmetric cell division and cortical reorganization in the mouse oocyte. Immunostaining showed that in fully grown germinal vesicle (GV) oocytes, ING3 localized predominantly in the GV. After germinal vesicle breakdown (GVBD), ING3 homogeneously localized in the cytoplasm. In oocytes where *Ing3* was targeted by siRNA microinjection, we observed symmetric cell division during mouse oocyte maturation. In those oocytes, oocyte polarization was not established due to the failure to form an actin cap or a cortical granule-free domain (CGFD), the lack of which inhibited spindle migration. These features were among the main causes of abnormal symmetric cell division. Interestingly, an analysis of the mRNA expression levels of genes related to asymmetric cell division revealed that only *mTOR* was downregulated, and, furthermore, that genes downstream of mTOR (*e.g.*, *Cdc42*, *Rac1*, and *RhoA*) were also downregulated in si*Ing3*-injected oocytes. Therefore, ING3 may regulate asymmetric cell division through the *mTOR* pathway during mouse oocyte maturation.

## Introduction

Oocyte maturation in mammals is characterized by a unique asymmetric cell division. After germinal vesicle breakdown (GVBD), the centrally positioned spindle migrates to the oocyte cortex; thereafter, asymmetric cell division occurs. Accordingly, the oocyte is transformed into a highly polarized, large metaphase II (MII)-arrested oocyte with an extruded small polar body [1]. Failure of this asymmetric cell division to occur is usually observed in low quality oocytes or those that have experienced post-ovulatory aging, a cause of mammalian infertility [2], [3]. Asymmetric cell division depends upon the position of the spindle that is formed after GVBD. In a normal mouse oocyte, the germinal vesicle (GV) exists in the proximity of the central area of the oocyte [4], so that the spindle is formed near the center of the oocyte just after GVBD. Normal asymmetric cell division is induced after the spindle migrates to the oocyte cortex during meiotic maturation. It has been reported that spindle migration depends upon oocyte polarization, *e.g.,* the formation of an actin cap, where microfilaments are enriched, and a cortical granule free domain (CGFD), where cortical granules (CGs) are redistributed [5]–[9]. However, details of the molecular mechanisms underlying oocyte polarization are poorly understood.

During oocyte growth in the mouse, dynamic changes occur in chromatin structure and histone modifications. With respect to histones H3 and H4, the levels of methylated (H3K4) and acetylated lysine residues (H3K9, H3K18, H4K5, and H4K12), which are associated with active gene expression, increase during mouse oogenesis and peak in fully grown GV oocytes [10]. A previous study showed that deficiency of *Mll2,* a H3K4 methyltransferase, in mouse oocytes causes anovulation and oocyte death [11]. In *Mll2*-deficient oocytes, the levels of di- and trimethylated H3K4 (H3K4me2/3) are decreased, and abnormal maturation and aberrant gene expression, especially in apoptosis-related genes, are observed [11]. These results suggest that the levels of H3K4me2/3 in oocytes are important for mouse oocyte maturation. ING (inhibition of growth) family members contain a plant homeodomain (PHD) motif through which the proteins can bind to H3K4me3, and are highly conserved from yeast to humans [12]. ING family proteins have been found as subunits of chromatin remodeling complexes [13], and are involved in transcriptional regulation, DNA repair, tumorigenesis, apoptosis, cellular senescence, and cell cycle arrest [14], [15]. ING3 is an important subunit of the human NuA4 histone acetyltransferase complex [16]. ING3 is a tumor suppressor in melanoma and head and neck squamous cell carcinoma (HNSCC), and is involved in the regulation of p53-mediated transcription, cell cycle control, and apoptosis [17]–[19]. Previous studies have demonstrated that overexpression of *ING3* in the human colorectal cancer cell line, RKO, suppresses cell growth through cell cycle control and induces apoptosis in a p53-dependent manner; however, no direct interactions between ING3 and p53 have been confirmed by co-immunoprecipitation [17]. In melanoma cells, the overexpression of *ING3* induced *FAS* expression and promoted UV-induced apoptosis through a FAS/CASPASE-8-dependent pathway in a p53-independent manner [20]. Although *ING3* is ubiquitously expressed in mammalian tissues, it has been reported that *ING3* is more highly expressed in mouse, rhesus monkey, and human oocytes [21]. However, the role(s) of ING3 in oocytes is unknown.

In the present study, we investigated whether ING3 functions during mouse oocyte maturation. We found that the loss of ING3 function after siRNA treatment inhibited actin cap and CGFD formation, as well as oocyte polarization, leading to symmetric cell division. Since ING3 is involved in transcriptional regulation [14], [15], we also investigated whether ING3 regulated genes involved in asymmetric cell division during oocyte maturation. Interestingly, we found that among several asymmetric cell division–related genes, only *mTOR* expression was downregulated. Furthermore, mTOR’s downstream genes, the Rho-family small GTPases (*e.g.*, *Cdc42*, *Rac1*, and *RhoA*) were also downregulated in oocytes after siRNA injection. It has also been reported that GTPases, including Cdc42, Rac1, and RhoA, can activate actin polymerization [22]–[24]. Given these results, we propose that ING3 plays an important role in governing asymmetric cell division during mouse oocyte maturation by regulating actin polymerization via the *mTOR* pathway.

## Materials and Methods

### Oocyte Collection and Culture

Fully grown GV oocytes were collected from the ovaries of 8- to 10-week-old ICR mice (SLC, Shizuoka, Japan) in M2 medium containing 5.0 µM Milrinone (138-13801, Wako Pure Chemical Industries, Osaka, Japan). Milrinone, which increases cAMP, was used to inhibit GVBD. For maturation, oocytes were washed and cultured in M16 medium containing 4 mg/ml BSA (A311, Sigma-Aldrich, St. Louis, MO) under mineral oil (Sigma-Aldrich) at 37°C, in an atmosphere of 5% CO_2_ in air.

### Immunofluorescent Staining

Oocytes were collected for immunofluorescent staining after 0, 2, 8, 9.5, or 12 h in culture, at which point most of the oocytes had reached the fully grown GV, GVBD, MI, ATI, or MII stages, respectively. For α-tubulin and actin staining, oocytes were fixed in 4% paraformaldehyde (Sigma-Aldrich) in Phosphate Buffered Saline (PBS) for 30 min at room temperature (RT). After washing three times in PBS containing 0.3% polyvinylpirrolidone (PVP K-30, Nacalai Tesque, Kyoto, Japan; PBS/PVP), oocytes were treated with 0.5% Triton X-100 (Sigma-Aldrich) in PBS for 40 min at RT, blocked in PBS containing 1.0% BSA (A9647, Sigma-Aldrich; blocking solution) for 1 h at RT and incubated overnight at 4°C with a FITC-conjugated anti-α-tubulin antibody (1∶200 dilution; F2168, Sigma-Aldrich) or 2 µg/ml TRITC-conjugated phalloidin (P1951, Sigma-Aldrich) in blocking solution. For staining of ING3, cortical granules (CGs), acetylated H4K12 (AcH4K12), and mTOR, zona pellucidae were removed from the oocytes by acid Tyrode’s solution (pH 2.5) and oocytes were fixed in PBS containing 4% paraformaldehyde for 20 min at 4°C. After washing three times in PBS/PVP, oocytes were treated with 0.5% Triton X-100 in PBS for 40 min at RT, blocked in blocking solution for 1 h at RT, and incubated overnight at 4°C with one of the following reagents diluted into blocking solution: mouse anti-ING3 antibody (1∶100 dilution, sc-101245, Santa Cruz Biotechnology Inc., Dallas, TX); 2 µg/ml FITC-conjugated lectin (L7381, Sigma-Aldrich); rabbit anti-AcH4K12 antibody (1∶300 dilution 06-761, Millipore Corp., Billerica, MA); or rabbit anti-mTOR antibody (1∶2000 dilution, ab2732, Abcam Ltd., Cambridge, UK). For ING3, AcH4K12, and mTOR staining, oocytes were washed three times in blocking solution and then incubated in blocking solution containing the secondary antibody (Alexa Fluor 488–conjugated goat anti-mouse IgG, 1∶500 dilution, Invitrogen, Carlsbad, CA; Alexa Fluor 594–conjugated goat anti-rabbit IgG, 1∶500 dilution, Invitrogen; or Alexa Fluor 488–conjugated goat anti-rabbit IgG, 1∶500 dilution, Invitrogen) for 1 h at RT. After staining of α-tubulin, actin, ING3, CGs, AcH4K12, and mTOR, oocytes were washed three times in blocking solution for 15 min and nuclei were stained in PBS containing 10 µg/ml Hoechst 33342 (Sigma-Aldrich) for 10 min. After immunofluorescent staining, oocytes were mounted on slides in 50% glycerol/PBS and fluorescent signals were detected using a fluorescence microscope (BX50, Olympus, Tokyo, Japan). At least 20 oocytes were examined for each group.

### Triton Treatment of Oocytes Prior to PFA Fixation

Triton treatment prior to PFA fixation was performed in order to examine whether protein was bound to chromatin [26], [27]. Oocytes were treated with 0.2% Triton X-100 in PBS for 30 s. Then, they were washed with PBS three times and fixed in 4% PFA for 5 min at RT. After washing with PBS, immunofluorescent staining was performed as described above.

### *Ing3* siRNA Injection

Approximately 5–10 pl of 50 µM *Ing3* siRNA (si*Ing3*; RNAi Inc., Japan, 5′-GGAUGAGAGGCGUUUACGUGC-3′) in annealing buffer consisting of 30 mM HEPES-KOH (pH 7.4), 100 mM KOAc and 2 mM Mg(OAc)_2_ was microinjected into the cytoplasm of a fully grown GV oocyte. The same amount of negative control siRNA (siControl; RNAi Inc.), which contains scrambled sequences from the si*Ing3* construct, was also microinjected as a control. After injection, the oocytes were cultured in M16 medium containing 5.0 µM Milrinone and 4 mg/ml BSA for 15.5 h, and then washed five times in fresh M16 medium containing 4 mg/ml BSA. Then, the oocytes were transferred to fresh M16 medium containing 4 mg/ml BSA and cultured under mineral oil at 37°C in an atmosphere of 5% CO_2_ in air or harvested for quantitative RT-PCR (qRT-PCR) or immunoblotting.

### RNA Extraction and qRT-PCR

Total RNA from 50 fully grown GV oocytes was extracted using the TRIzol reagent (Invitrogen). RNase-free DNase I (Roche Diagnostics Corp., Indianapolis, IN) was added to the preparations to avoid genomic DNA contamination. For reverse transcription, Rever Tra Ace (Toyobo Co., Ltd., Osaka, Japan) and Oligo dT primer (Invitrogen) were used, according to the manufacturer’s instructions. qPCR was carried out with THUNDERBIRD qPCR Mix (Toyobo Co., Ltd.) using Rotor-Gene 6000 (Qiagen, Hilden, Germany). Transcription levels were determined three times and normalized to *Gapdh*; relative gene expression was analyzed using the 2^−ΔΔCt^ method [25]. All primers used for PCR are listed in Table 1.

**Table 1:** Primers used for qRT-PCR.

Genes	GenBank Accession No.	Forward	Reverse
*Ing3*	NM_023626.4	ttcacatactcccgtggaaaa	gcgcttcagatttgaatttctt
*Arpc3*	NM_019824.3	gaaggaaatgtacacgctaggaa	gaggtacgcacgcatcatc
*JMY*	NM_021310.3	ttcaaattacaagccgtgcacccg	agctgccttctggacctttactga
*mTOR*	NM_020009.2	cttggagaaccagcccataa	ctggtttcaccaaaccgtct
*ARF1*	NM_001130408.1	Atgcgcattctcatggtg	aacagtctccacattgaaacca
*Hsp90α*	NM_010480.5	ggagataaatcctgatcactcca	caagatgaccagatccttcaca
*Fmn2*	NM_019445.2	gagacccttcaagctctctatga	gaagatcgactgtgcttttcaa
*Mos*	NM_020021.2	aagggaaaggaactgggatg	aacagccagggaagtttgg
*Whamm*	NM_001004185.3	cagccatttagagacatgcgagaa	ctaggacccagctcatcctcatc
*Cdc42*	NM_001243769.1	ttgttggtgatggtgctgtt	aatcctcttgccctgcagta
*Rac1*	NM_009007.2	agatgcaggccatcaagtgt	gagcaggcaggttttaccaa
*RhoA*	NM_016802.4	acaactgcatcccagaacct	taccacaagctccatcacca

### Immunoblotting

Fifty fully grown GV oocytes were collected in SDS sample buffer and boiled at 95°C for 4 min. The samples were kept at −80°C until use. Total protein was separated by 12.5% SDS-polyacrylamide gel electrophoresis (SDS-PAGE) for 90 min at 20 mA and electrophoretically transferred to a polyvinylidene fluoride (PVDF) membrane (Immobilon-P, Millipore, Bedford, MA) over 2 h at 50 V. After three washes in Tris buffered saline (TBS) containing 0.1% Tween-20 (TBST), the membrane was blocked in TBST containing 5% skim milk for 1 h, and then incubated with either a mouse anti-ING3 antibody (1∶200 dilution) or a mouse anti-α-tubulin antibody (1∶5000 dilution; T9026; Sigma-Aldrich) in TBST containing 2% skim milk overnight at 4°C. After three washes in TBST, the membrane was incubated with an HRP-conjugated anti-mouse secondary antibody (1∶2000 or 1∶10000 dilution; GE Healthcare UK Ltd, Amersham, UK) in TBST for 1 h at RT. The membrane was extensively washed three times with TBST, and then processed with the Enhanced Chemilumidescence (ECL) detection system (GE Healthcare UK Ltd). α-tubulin was detected as an internal control.

### Ethical Approval for the Use of Animals

All animal experiments were approved by the Animal Research Committee of Kyoto University (Permit Number: 24-17) and were performed in accordance with the committee’s guidelines.

### Statistical Analysis

Each experiment was repeated at least three times. The values were analyzed using a *t*-test. *p* values <0.05 were considered to be statistically significant.

## Results

### Localization of ING3 during Oocyte Maturation in the Mouse

**Figure 1:**
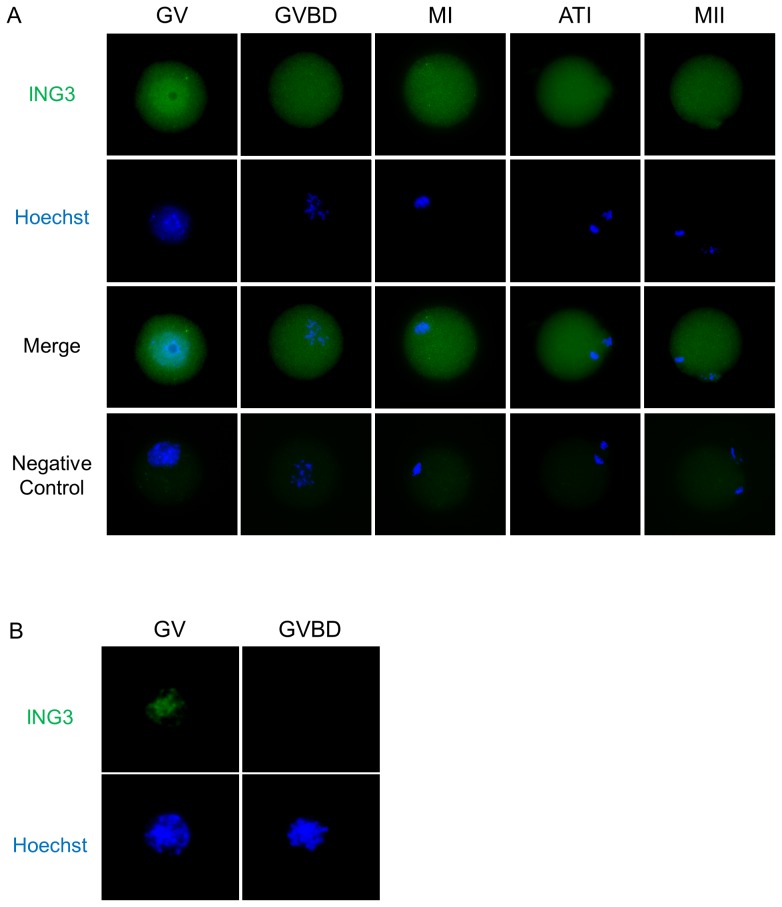
Localization of ING3 during mouse oocyte maturation. (A) ING3 was predominantly localized in the nucleus in fully grown GV oocytes. After GVBD, ING3 localized homogeneously throughout the cytoplasm. Green, ING3; blue, chromatin. (B) ING3 bound to the chromatin in fully grown GV oocytes. Green, ING3; blue, chromatin.

To investigate the role(s) of ING3 during mouse oocyte maturation, ING3 localization was initially examined at different stages of meiotic maturation. In the fully grown GV oocyte, ING3 was localized in GV. After GVBD, ING3 was dispersed homogenously into the cytoplasm (Fig. 1). Furthermore, ING3 bound to the chromatin in fully grown GV oocytes (Fig. 1B).

### Loss of ING3 Function Leads to Spindle Migration Failure and the Formation of a Large Polar Body

**Figure 2:**
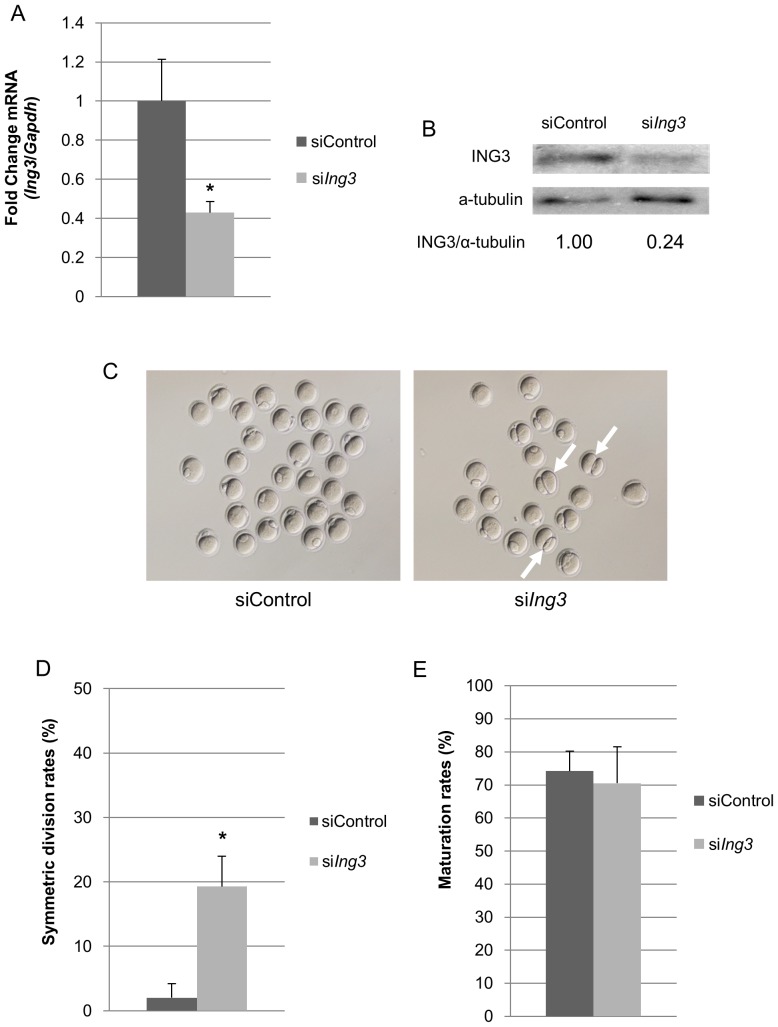
Effects of si*Ing3* injection on asymmetric cell division in mouse oocytes. (A) *Ing3* mRNA was significantly decreased in the fully grown GV oocytes injected with si*Ing3* (*p<0.05). (B) ING3 protein levels, after normalization to α-tubulin, were decreased in fully grown GV oocytes injected with si*Ing3*. (C) Abnormal cell division was observed in several si*Ing3*-injected oocytes at the MII stage (arrows). (D) In si*Ing3*-injected oocytes, the rate of symmetric division was significantly increased as compared to that observed in siControl-injected oocytes (*p<0.05). (E) Maturation rates were not different between si*Ing3*- and siControl-injected oocytes.

**Figure 3:**
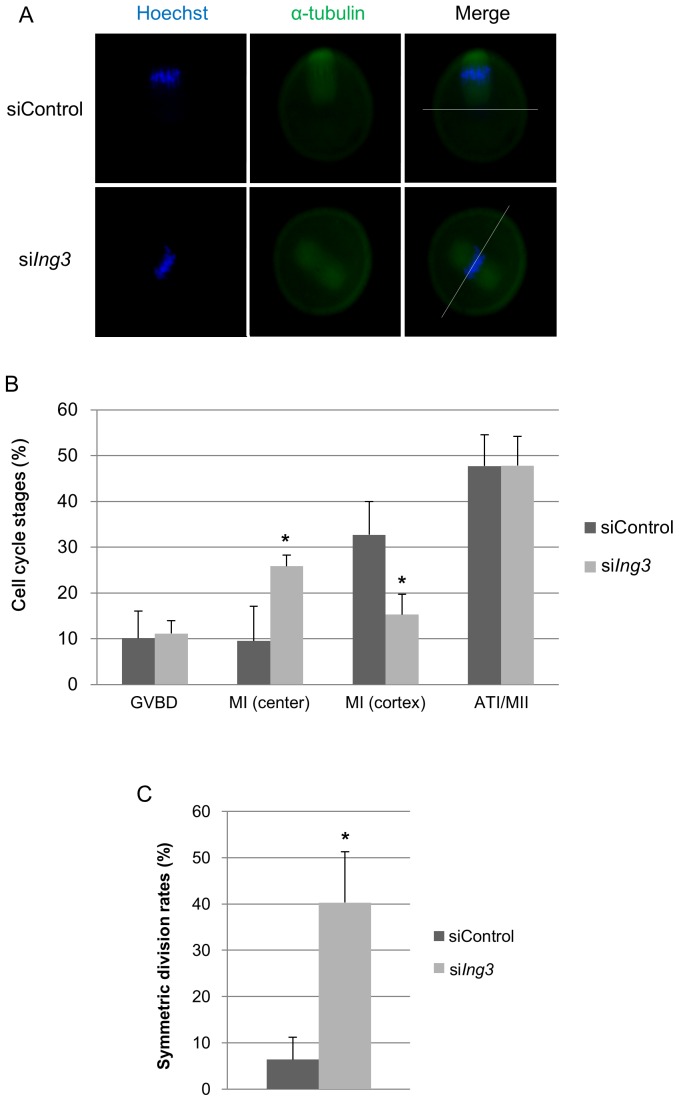
Effects of si*Ing3* injection on spindle migration in mouse oocytes. (A) In siControl-injected oocytes, the spindle is located near the cortex at the MI stage after 9.5 h in culture. By contrast, in si*Ing3*-injected oocytes, the spindle is located at the center of the oocyte at the MI stage after 9.5 h in culture. Bars indicate the central position of the oocyte. Green, α-tubulin; blue, chromatin. (B) In siControl-injected oocytes, the frequency of spindles located at the cortex was significantly increased. By contrast, in si*Ing3*-injected oocytes, the frequency of spindles located at the center of the oocytes was significantly increased (*p<0.05). The oocytes that were extruding or had extruded a polar body were considered to be at the ATI or MII (ATI/MII) stage. (C) In si*Ing3*-injected oocytes, the rate of symmetric division was significantly increased at the ATI/MII stages (*p<0.05).

The results of qRT-PCR and immunoblotting showed that the amount of *Ing3* mRNA and protein was dramatically decreased in si*Ing3*-injected oocytes (Fig. 2A, B). After maturation in culture, si*Ing3*-injected oocytes exhibited symmetric cell division, showing 2-cell-like MII oocytes. Furthermore, the rate of symmetric cell division was significantly higher than that observed in siControl-injected oocytes. However, polar body (PB) extrusion was observed in most of the population (Fig. 2C–E). To analyze the phenotype of si*ing3*-injected oocytes, spindle migration was confirmed by immunostaining of α-tubulin. After 9.5 h in culture, the spindles moved to the cortex of the oocytes in siControl-injected oocytes, but remained centrally located in si*Ing3*-injected oocytes (Fig. 3A, B). Moreover, the rate of symmetric cell division was dramatically increased at the end of the first meiosis in si*Ing3*-injected oocytes (Fig. 3C).

### Loss of ING3 Function Abrogates Cortical Reorganization

**Figure 4:**
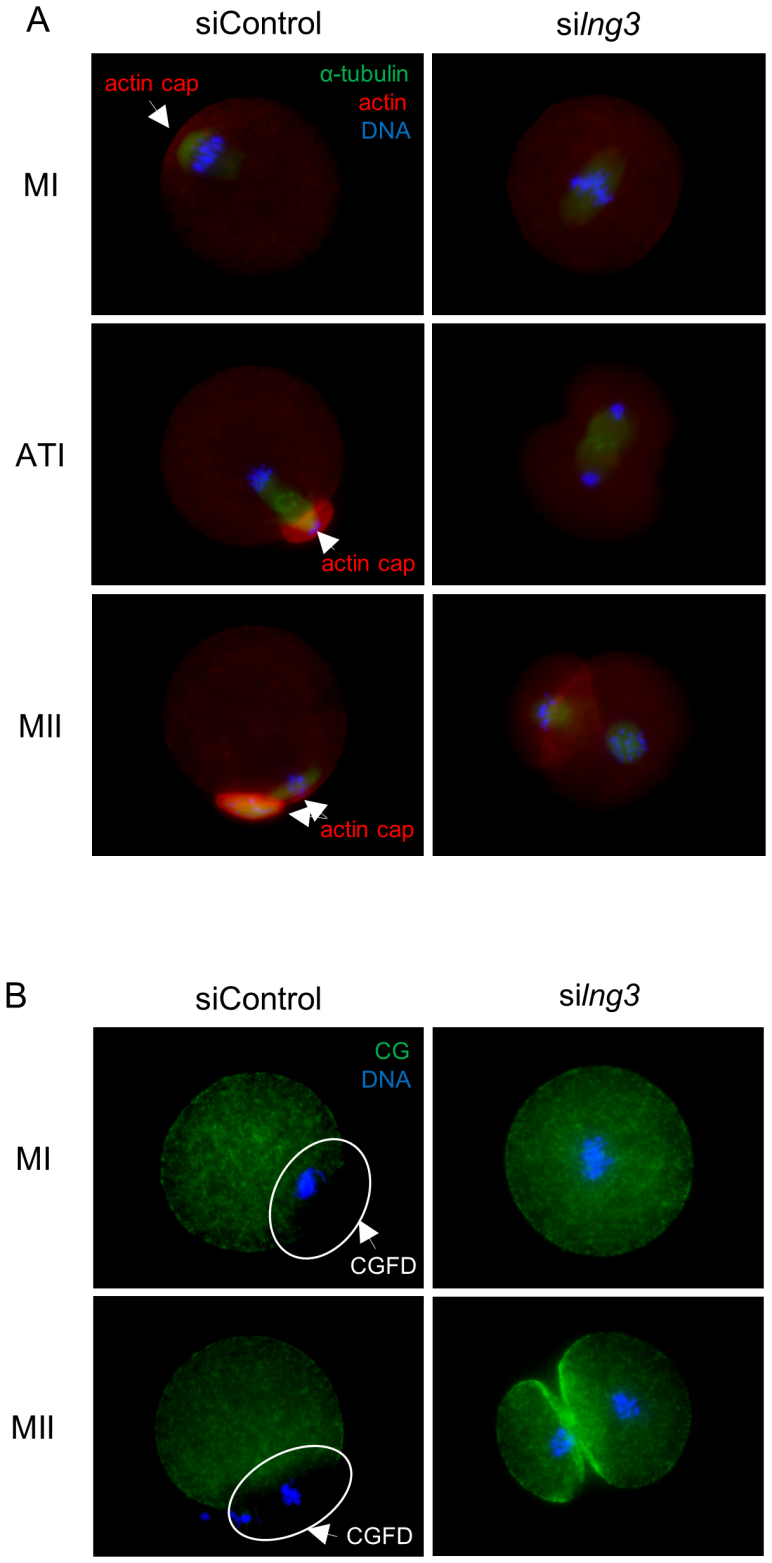
Effects of si*Ing3* injection on cortical reorganization in mouse oocytes. (A) Actin cap formation was noted in siControl-injected oocytes at the MI, ATI, and MII stages. By contrast, no actin cap was formed in si*Ing3*-injected oocytes at any stage. Arrows indicate the actin cap. Green, α-tubulin; red, actin; blue, chromatin. (B) CGs were absent in the cortex where the chromosomes were located at the MI and MII stages in siControl-injected oocytes. By contrast, in si*Ing3*-injected oocytes, CGs were distributed throughout the cortex at the MI and MII stages, and were intensely localized in the region of cell adhesion at the MII stage. Circles and arrows denote the CGFD. Green, cortical granules; blue, chromatin.

Since spindle migration and asymmetric cell division are intimately related to oocyte polarity, the formation of the actin cap and CGFD, predominant features of oocyte polarization, was examined. As shown in Fig. 4A, in siControl-injected oocytes the chromosomes had already moved to the cortical layer, the site of actin cap formation, by the MI stage after 9.5 h in culture, and at the ATI stage, the chromosomes dissociated at the region of the cortex with the actin cap. By the MII stage, a small PB and a large MII oocyte had formed and the chromosomes were located under the region of the cortex where the actin cap formed. By contrast, in si*Ing3*-injected oocytes, the chromosomes had not moved from the center of the cytoplasm and no actin cap was observed at the MI stage after 9.5 h in culture, and at the ATI stage, the chromosomes dissociated around the center of the cytoplasm. By the MII stage, an MII oocyte formed a 2-cell-like structure with no actin cap. As shown in Fig. 4B, in siControl-injected oocytes, CGs were lacking near the cortical layer where the chromosomes were located at the MI and MII stages, whereas in si*Ing3*-injected oocytes the chromosomes had not moved from the center of the cytoplasm and CGs were widely distributed through the cortical layer at the MI stage and were intensely localized in the cell adhesion region at the MII stage.

### ING3 Regulates *mTOR* Expression in GV Oocytes

**Figure 5:**
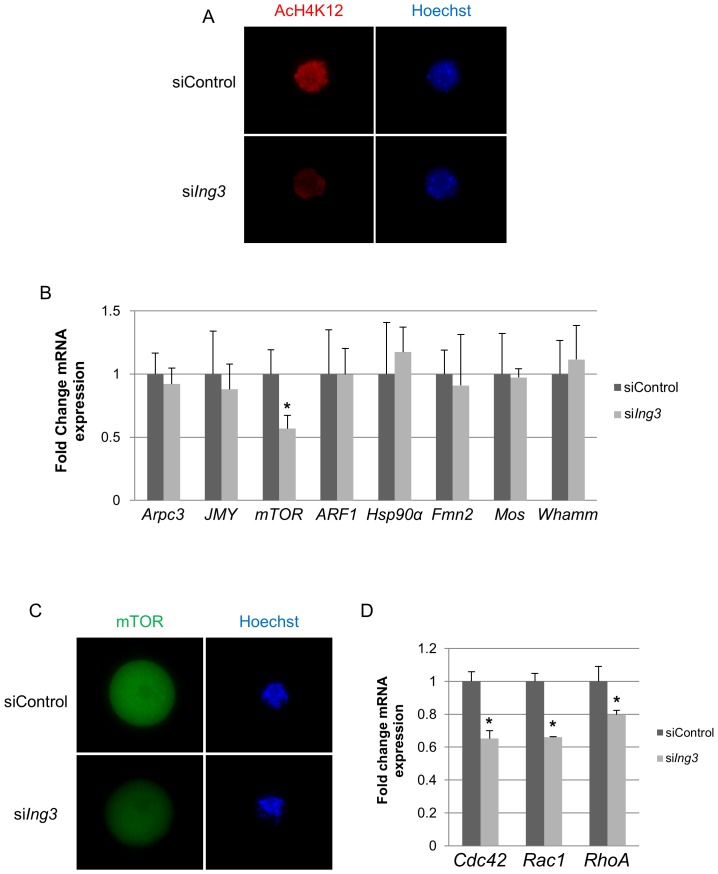
Effects of si*Ing3* injection on the levels of AcH4K12, asymmetric cell division–related gene expression, and mTOR protein in mouse oocytes. (A) The levels of AcH4K12 in fully grown GV oocytes cultured for 15.5 h after injection of si*Ing3* were significantly decreased as compared to controls. Red, AcH4K12; blue, chromatin. (B) *mTOR* mRNA levels were significantly decreased after si*Ing3* injection in fully grown GV oocytes (*p<0.05) as compared with other asymmetric cell division–related genes. (C) Although the protein localization of mTOR was not changed, the amount of mTOR protein was reduced in si*Ing3*-injected oocytes. Green, mTOR; blue, chromatin. (D) The expression levels of mTOR-downstream genes, such as *Cdc42*, *Rac1*, and *RhoA,* in fully grown GV oocytes were significantly decreased 15.5 h after si*Ing3* injection (*p<0.05).

The proportion of AcH4K12 that is acetylated by the NuA4 histone acetyltransferase complex [13] in the fully grown GV oocyte was confirmed by immunofluorescent staining. In si*Ing3*-injected oocytes, the levels of AcH4K12 were decreased (Fig. 5A). To investigate the influence of this reduction in AcH4K12 levels in fully grown GV oocytes, the expression levels of the genes involved in asymmetric cell division were examined. In si*Ing3*-injected oocytes, the expression of *mTOR* was significantly downregulated as compared with the expression of *Arpc3*, *JMY*, *ARF1*, *Hsp90α*, *Fmn2*, *Mos*, or *Whamm* (Fig. 5B). Furthermore, immunostaining and qRT-PCR data showed that mTOR protein levels and mRNA levels of Rho-family GTPases, such as *Cdc42*, *Rac1*, and *RhoA,* were also decreased in si*Ing3*-injected oocytes (Fig. 5C, D).

## Discussion

ING3, which is an important subunit of the human NuA4 histone acetyltransferase complex and can recognize H3K4me3, is mainly known as a tumor suppressor gene. ING family proteins are involved in chromatin remodeling and regulate transcription [14]. ING3 activates p53-transactivated promoters and modulates p53-mediated transcription, cell cycle control, and apoptosis [17]. Here, we observed the subcellular localization of ING3 at different stages of meiotic maturation and the binding of ING3 to the chromatin in fully grown GV oocytes. Together with the knowledge that ING3 is an important subunit of the NuA4 histone acetyltransferase complex, these observations suggested that ING3 functions as a chromatin remodeling factor in fully grown GV oocytes. We also found that loss of ING3 function led to symmetric cell division due to a failure in spindle migration. Asymmetric cell division depends on the position of the spindle that is formed after GVBD. The maintenance of a central spindle position leads to symmetric cell division, a feature observed in aging mouse oocytes [2], [3], [26]. Spindle migration is mainly regulated by actin filaments during oocyte maturation [1], [5], [6], and therefore is blocked by the inhibition of actin polymerization. In oocytes treated with cytochalasin D, an inhibitor of actin polymerization, spindle migration does not occur [27]. During mitosis of somatic cells, actin filaments are formed with polymerized actin and actin polymerization is regulated by the interaction of proteins called nucleation promoting factors (NPFs) with the Arp2/3 complex [28]. NPFs initially interact with an actin monomer and polymerize actins to induce the formation of unbranched actin filaments. NPFs, actin filaments, and an actin monomer cooperate to stimulate the inactive Arp2/3 complex to form actin filament branches on the side of existing filaments [29]. During mouse oocyte maturation, Arp2/3 co-localizes with the actin cap. This subcellular localization is changed in oocytes treated with cytochalasin B, which is also an inhibitor of actin polymerization, and the loss of function of the *Arp2/3* complex causes symmetric cell division because the actin cap does not form where actin filaments are converged by phosphatidylinositol (3,4,5)-triphosphate (PtdIns(3,4,5)P3) [30], [31]. Together, these results suggest that actin polymerization is a key regulator of asymmetric cell division in the mouse. In addition, actin polymerization is also involved in oocyte polarization through formation of the actin cap. Oocyte polarization, as indicated by the formation of the actin cap and CGFD, is one of the most important processes underlying asymmetric cell division during oocyte maturation [7]–[9], and the disruption of oocyte polarization results in symmetric cell division [31]–[35]. Thus, the disruption of oocyte polarization in *Ing3*-inhibited oocytes abrogated spindle migration and led to symmetric cell division.

It has been reported that the loss of function of *Arp2/3*, *JMY*, *mTOR*, *ARF1*, *Hsp90α, GM130*, *Fmn2*, *Mos*, or *Whamm* during mouse oocyte maturation disrupts oocyte polarity, impedes spindle migration, and leads to symmetric cell division [31]–[39]. Since ING3 is an important subunit of the NuA4 histone acetyltransferase complex, which activates gene expression, and ING3 bound to the chromatin in the fully grown GV oocytes, it is possible that ING3 regulates some genes that are involved in asymmetric cell division during oocyte maturation through histone acetylation. Indeed, we found that ING3 regulated the expression of *mTOR* and is involved in histone acetylation in fully grown GV oocytes, suggesting that ING3 regulates the expression of *mTOR* through histone acetylation. During mitosis in somatic cells, mTOR regulates actin polymerization by activating small GTPases (Cdc42, Rac1, and RhoA). In turn, activated small GTPases regulate the actin cytoskeleton and microtubule dynamics through the activation of NPFs [22], [23]. Cdc42, Rac1, and RhoA cooperate temporally and spatially to regulate actin dynamics and have an influence on cellular processes including cell motility, cell polarization, cell adhesion, and cytokinesis [24]. During oocyte maturation, mTOR also regulates oocyte polarization through its involvement in actin cap formation via the activation of small GTPases. Therefore, loss of mTOR function leads to symmetric cell division [32]. Thus, the observation that ING3 regulates the expression of *mTOR* and Rho-family small GTPases in fully grown GV oocytes suggests that ING3 plays important roles in actin polymerization via the *mTOR* pathway during mouse oocyte maturation.

Nonetheless, our results were slightly different from those obtained in mTOR inhibited oocytes. Namely, in mTOR-inhibited oocytes during mouse oocyte maturation cell cycle stages are delayed after 9.5 h in culture [32]. By contrast, loss of ING3 function did not delay the progression of cell cycle stages over 9.5 h in culture; however, the rate of symmetric cell division in oocytes at the end of first meiosis was significantly increased. It is probable that cell cycle regulation by RhoA proteins [40] was not sufficiently abrogated after microinjection of si*Ing3* in the fully grown GV oocytes despite the decrease observed in *RhoA* mRNA. This may explain the lack of notable effect on the cell cycle in these oocytes.

In eukaryotic cells, chromatin serves as a dynamic and active participant in multiple nuclear processes, and plays a remarkable role in the regulation of gene expression. Many histone methyltransferases and chromatin remodeling factors have pivotal roles in biological processes during development and disease, *e.g.,* neurological disorders and cancer [16], [41]. In addition, it has been reported that the levels of di- and trimethylated H3K4 in oocytes are important for maturation in the mouse [11], [42]. In oocytes lacking functional *Mll2*, the levels of di- and trimethylated lysine are decreased, chromosomes are misaligned, and maturation is arrested [11], suggesting that histone methyltransferase is important for meiosis. However, the roles of genes that govern histone methylation during mouse oocyte maturation are poorly understood. It has been reported that ING3 is a member of the ING family of proteins that bind to H3K4me3, and that ING3 is an important subunit of the human NuA4 histone acetyltransferase complex [13], [16], [43]. The NuA4 histone acetyltransferase complex governs the acetylation of histones H4 and H2A [16]. In this study, we found that ING3 was involved in the asymmetric cell division of mouse oocytes during the first meiotic cell division, and that ING3 promoted H4K12 acetylation, suggesting that the chromatin remodeling factors involved in the recognition of methylated H3K4 also function during mouse oocyte maturation.

In conclusion, our results demonstrated that ING3 function is critical for maintaining asymmetric cell division, and indicated the possibility that an elucidation of the genes involved in chromatin remodeling may shed light on the molecular mechanisms of mouse oocyte maturation.
